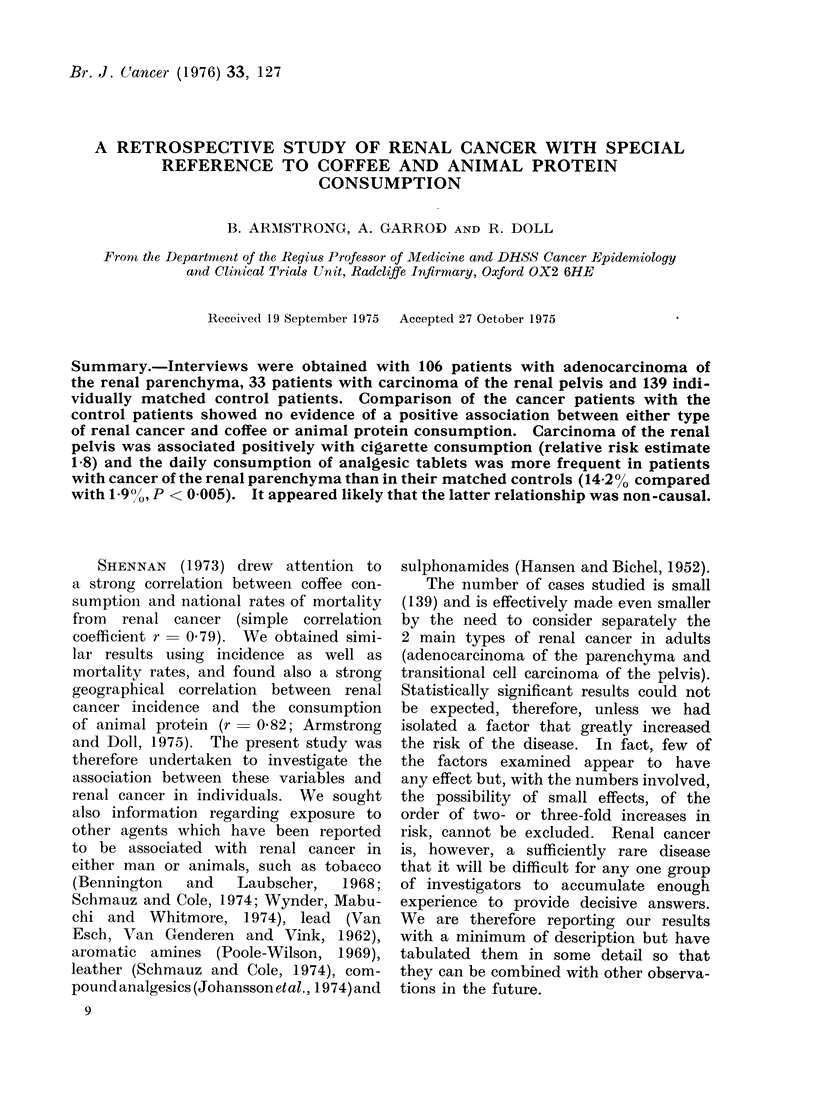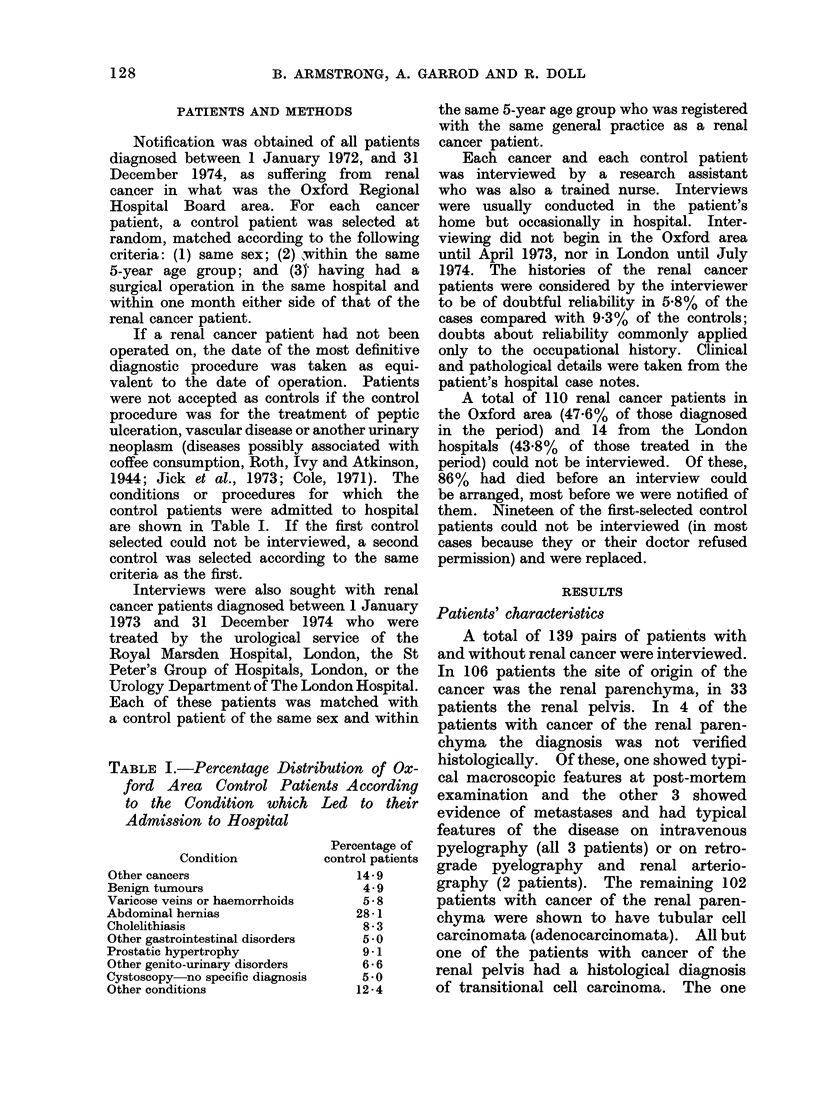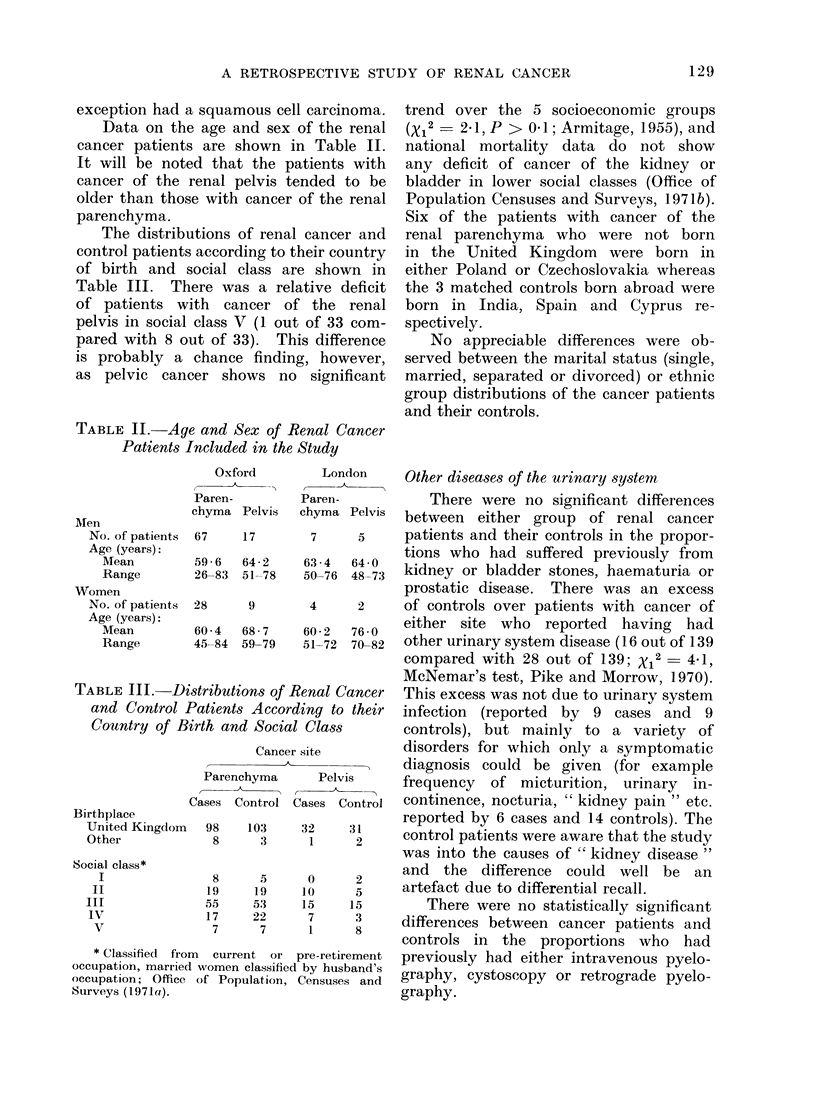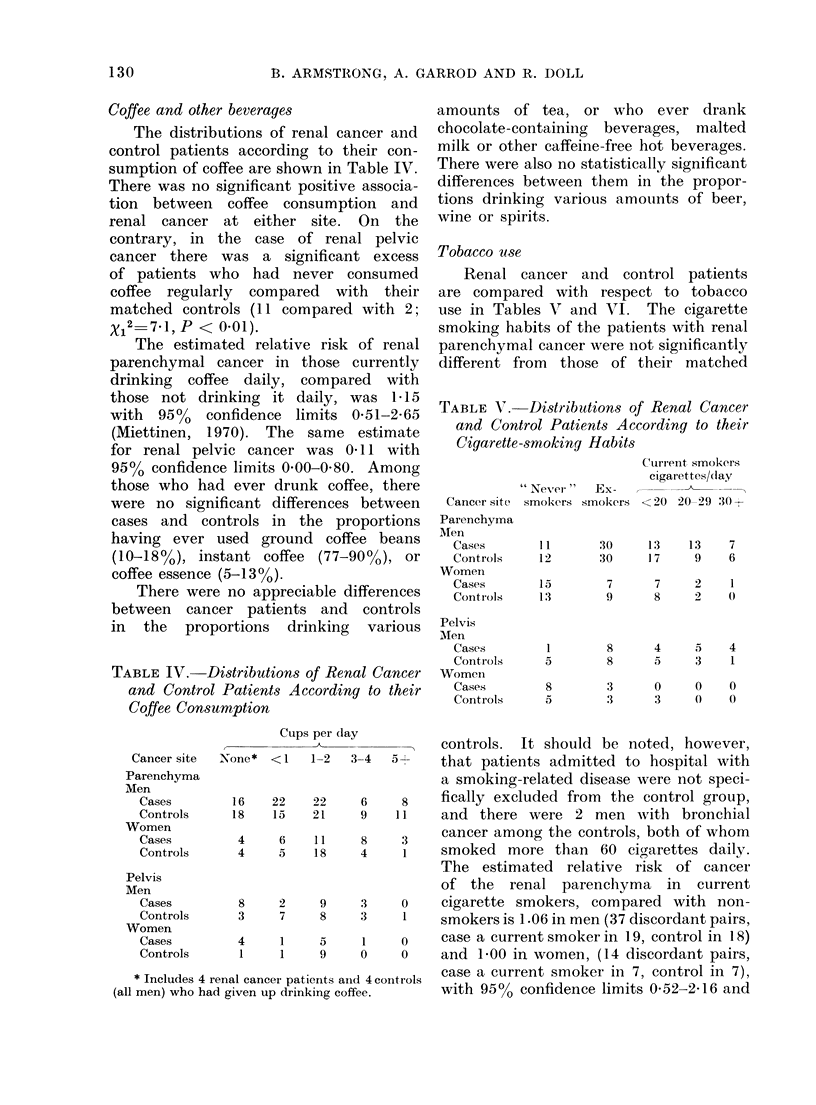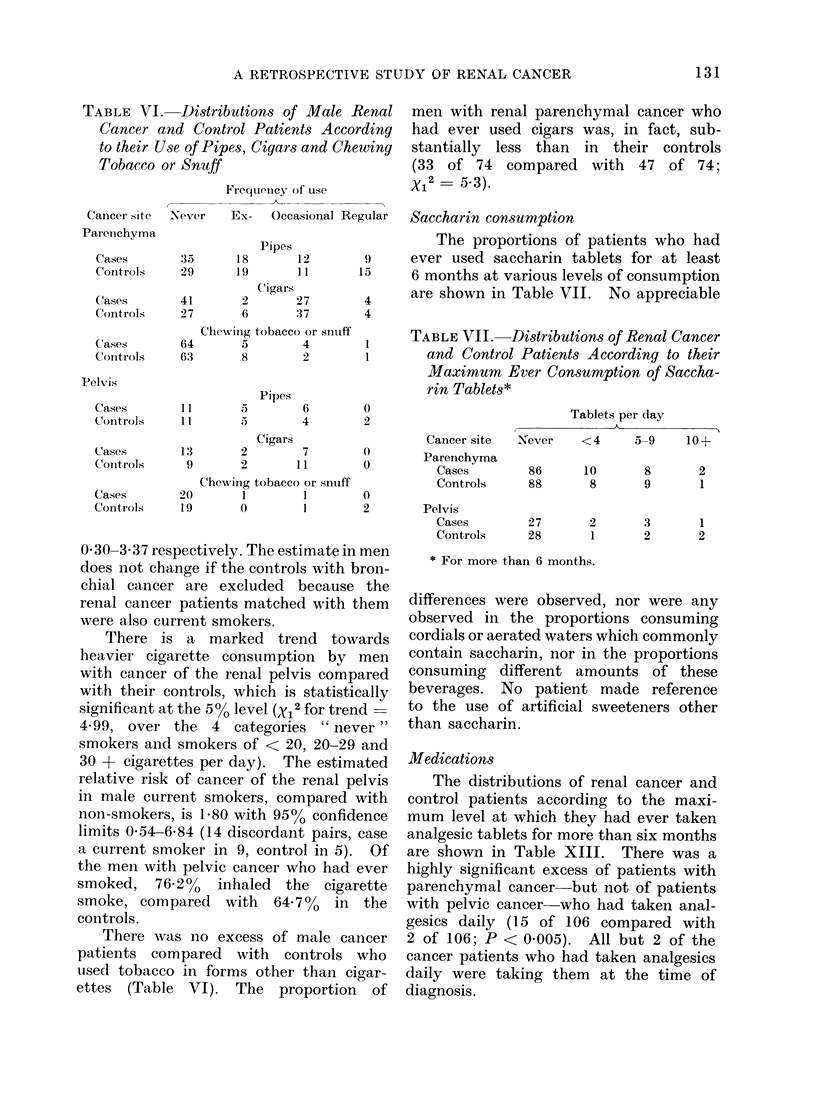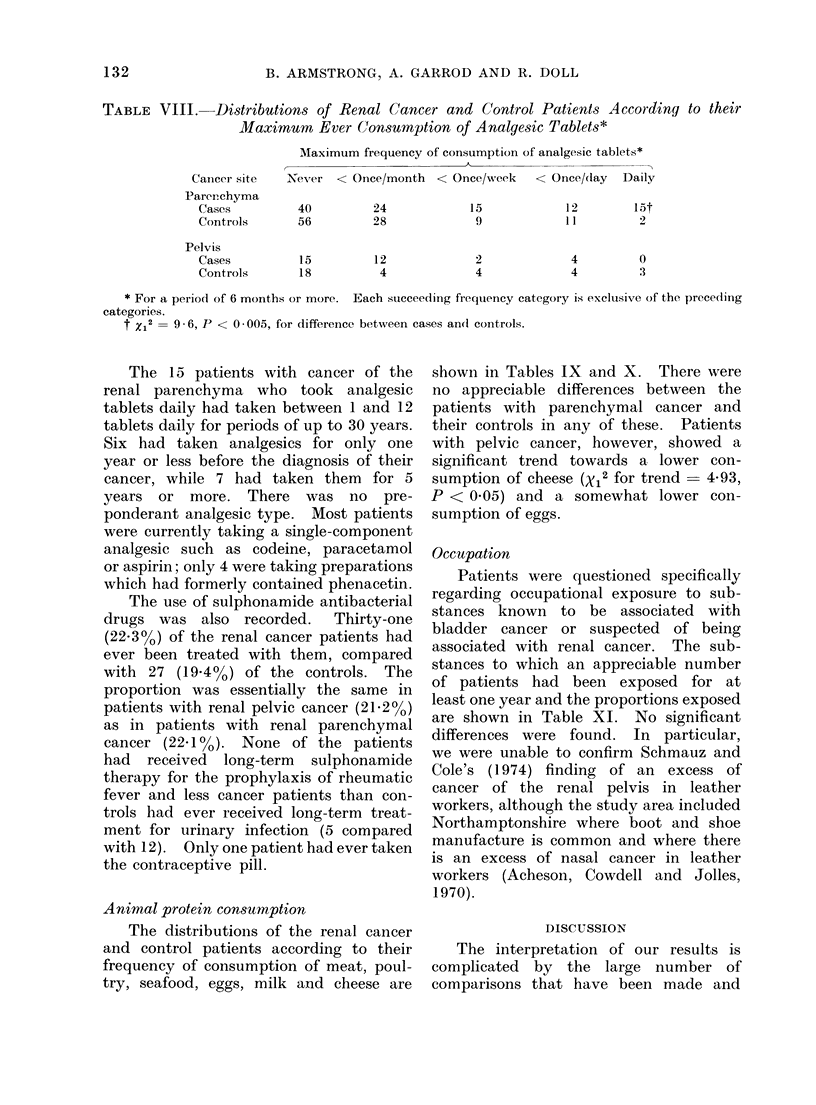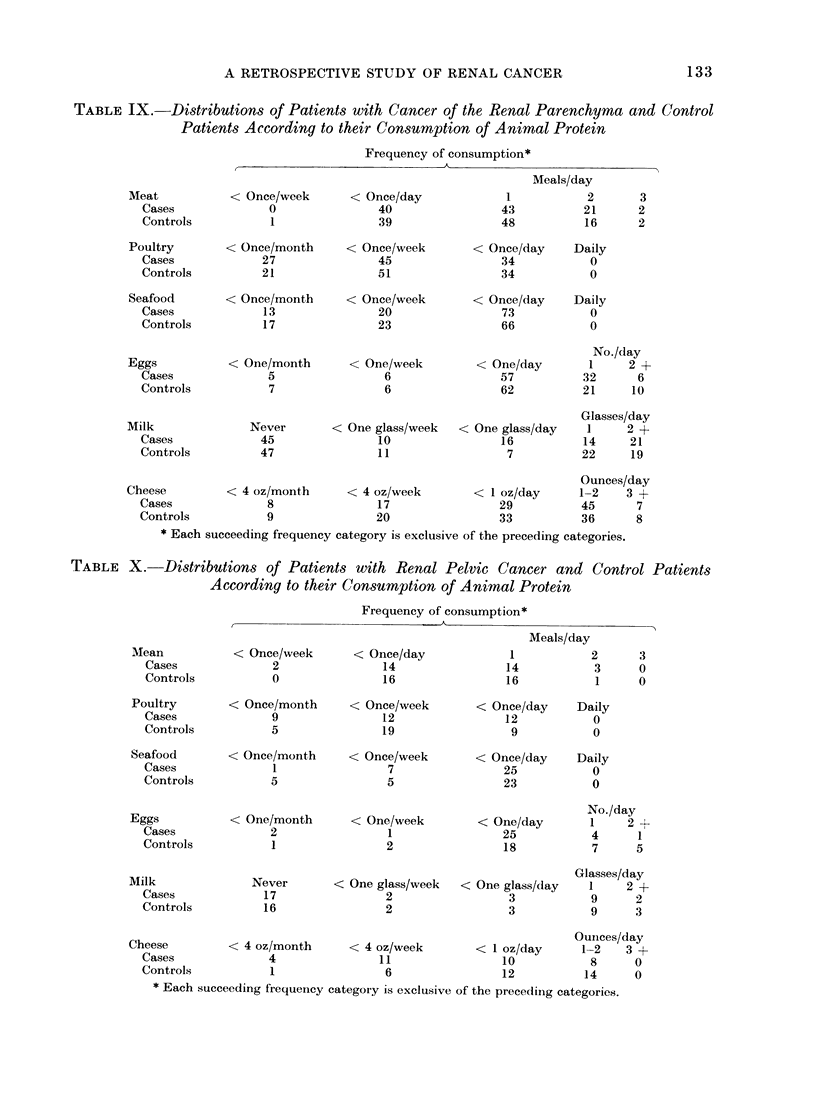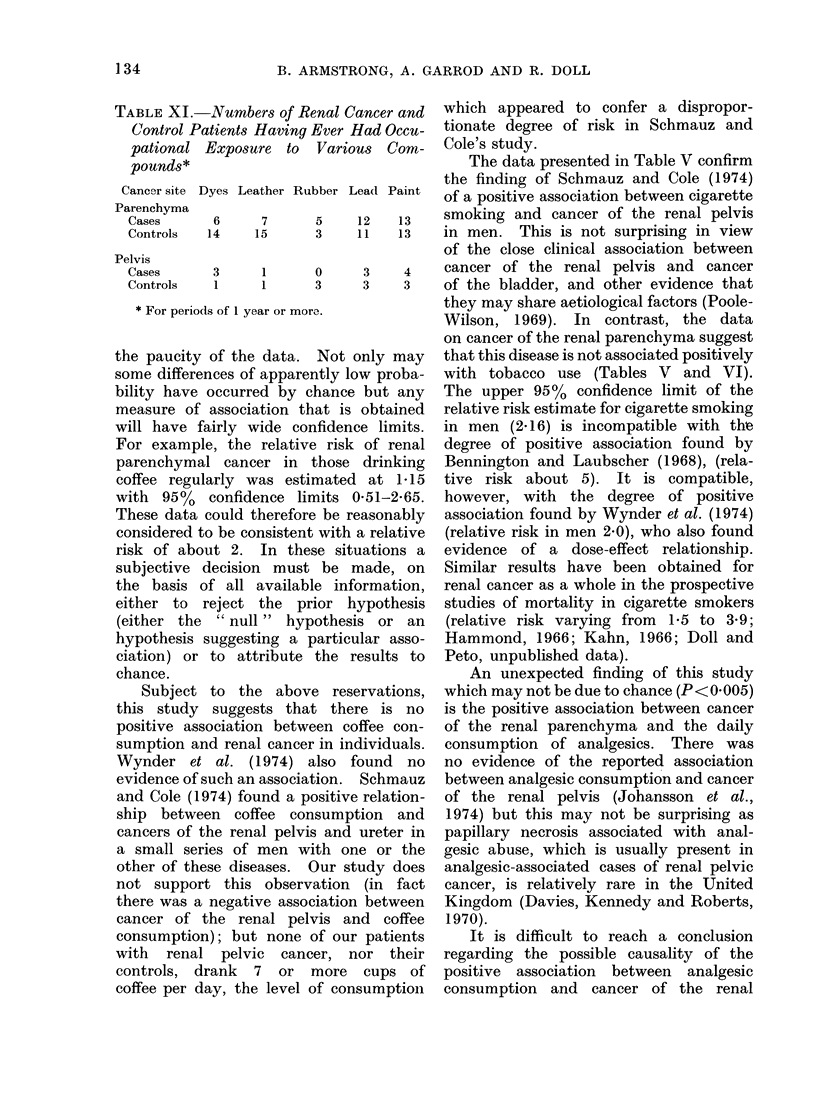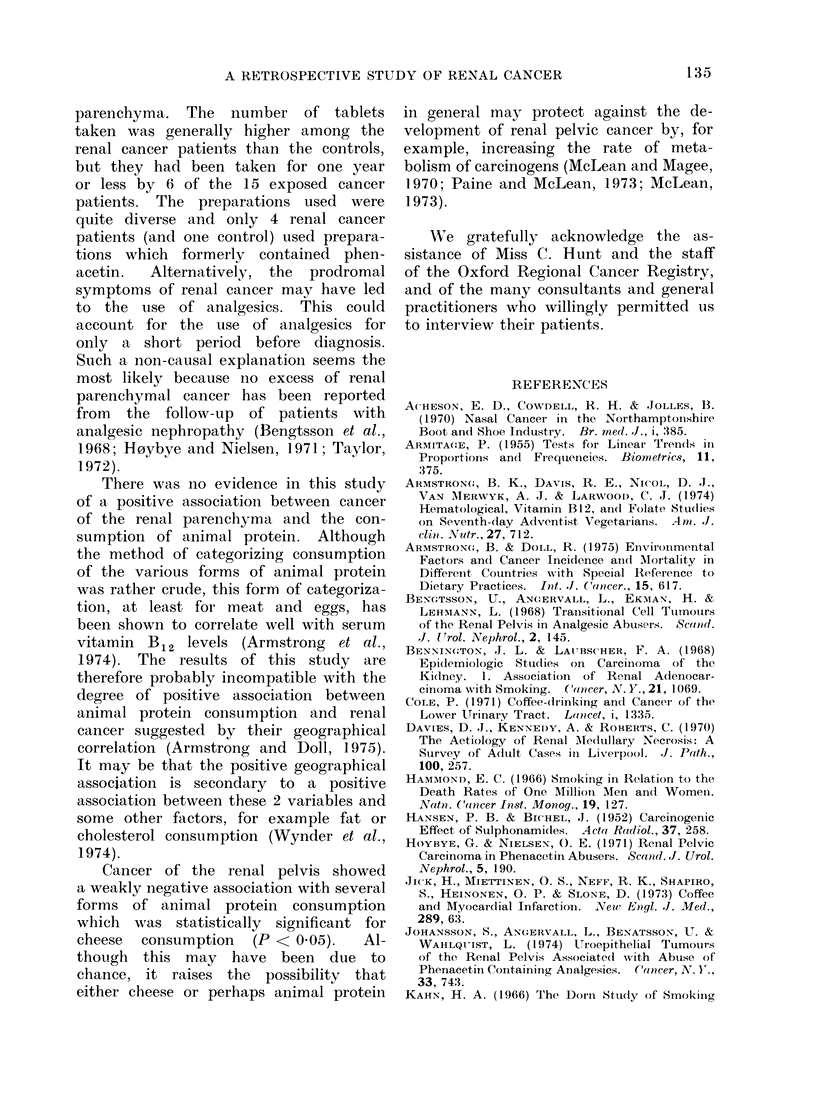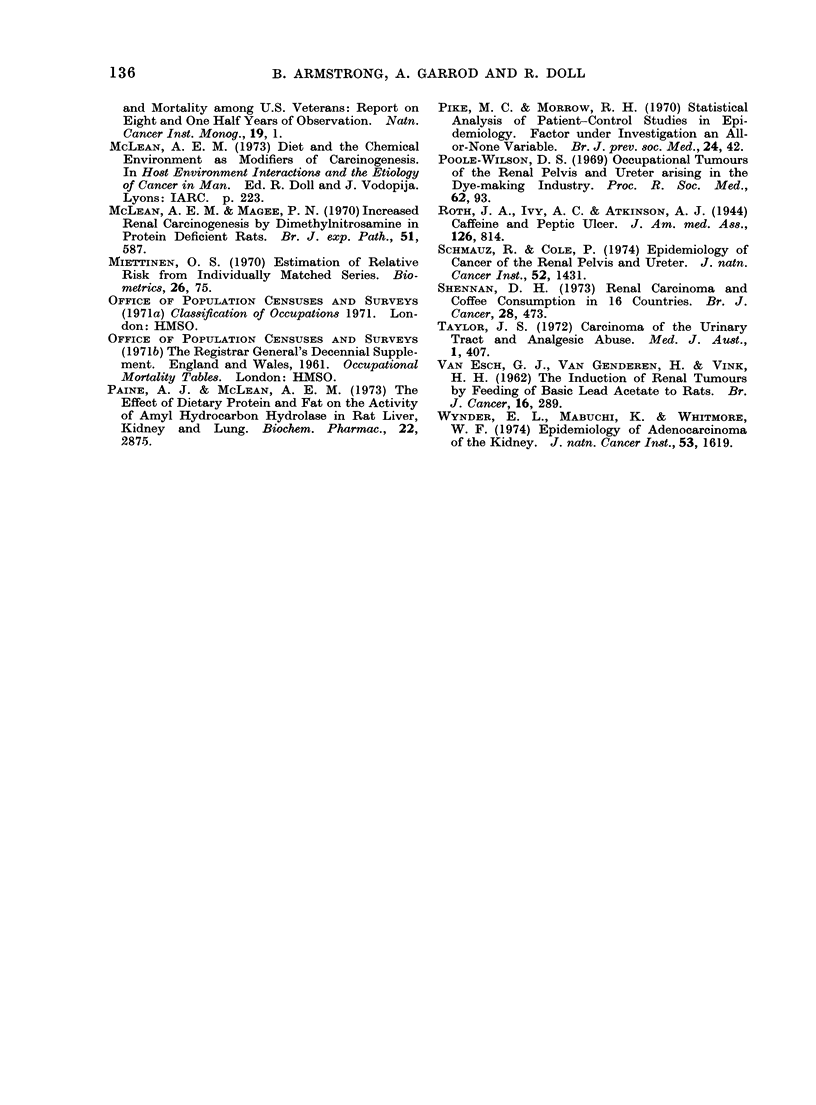# A retrospective study of renal cancer with special reference to coffee and animal protein consumption.

**DOI:** 10.1038/bjc.1976.17

**Published:** 1976-02

**Authors:** B. Armstrong, A. Garrod, R. Doll

## Abstract

Interviews were obtained with 106 patients with adenocarcinoma of the renal parenchyma, 33 patients with carcinoma of the renal pelvis and 139 individually matched control patients. Comparison of the cancer patients with the control patients showed no evidence of a positive association between either type of renal cancer and coffee or animal protein consumption. Carcinoma of the renal pelvis was associated positively with cigareete consumption (relative risk estimate 1-8) and the daily consumption of analgesic tablets was more frequent in patients with cancer of the renal parenchyma than in their matched controls (14-2% compared with 1-9%,P less than 0.005). It appeared likely that the latter relationship was non-causal.


*Br. J. Cancer* (1976) **33**, 127

### A RETROSPECTIVE STUDY OF RENAL CANCER WITH SPECIAL REFERENCE TO COFFEE AND ANIMAL PROTEIN CONSUMPTION

B. ARMSTRONG, A. GARROD AND R. DOLL

*From the Department of the Regius Professor of Medicine and DHSS Cancer Epidemiology and Clinical Trials Unit, Radcliffe Infirmary, Oxford OX2 6HE*

Received 19 September 1975 Accepted 27 October 1975

**Summary.—Interviews were obtained with 106 patients with adenocarcinoma of the renal parenchyma, 33 patients with carcinoma of the renal pelvis and 139 individually matched control patients. Comparison of the cancer patients with the control patients showed no evidence of a positive association between either type of renal cancer and coffee or animal protein consumption. Carcinoma of the renal pelvis was associated positively with cigarette consumption (relative risk estimate 1·8) and the daily consumption of analgesic tablets was more frequent in patients with cancer of the renal parenchyma than in their matched controls (14·2% compared with 1·9%, $P < 0{\cdot}005$). It appeared likely that the latter relationship was non-causal.**

SHENNAN (1973) drew attention to a strong correlation between coffee consumption and national rates of mortality from renal cancer (simple correlation coefficient $r = 0{\cdot}79$). We obtained similar results using incidence as well as mortality rates, and found also a strong geographical correlation between renal cancer incidence and the consumption of animal protein ($r = 0{\cdot}82$; Armstrong and Doll, 1975). The present study was therefore undertaken to investigate the association between these variables and renal cancer in individuals. We sought also information regarding exposure to other agents which have been reported to be associated with renal cancer in either man or animals, such as tobacco (Bennington and Laubscher, 1968; Schmauz and Cole, 1974; Wynder, Mabuchi and Whitmore, 1974), lead (Van Esch, Van Genderen and Vink, 1962), aromatic amines (Poole-Wilson, 1969), leather (Schmauz and Cole, 1974), compound analgesics (Johansson *et al.*, 1974) and sulphonamides (Hansen and Bichel, 1952).

The number of cases studied is small (139) and is effectively made even smaller by the need to consider separately the 2 main types of renal cancer in adults (adenocarcinoma of the parenchyma and transitional cell carcinoma of the pelvis). Statistically significant results could not be expected, therefore, unless we had isolated a factor that greatly increased the risk of the disease. In fact, few of the factors examined appear to have any effect but, with the numbers involved, the possibility of small effects, of the order of two- or three-fold increases in risk, cannot be excluded. Renal cancer is, however, a sufficiently rare disease that it will be difficult for any one group of investigators to accumulate enough experience to provide decisive answers. We are therefore reporting our results with a minimum of description but have tabulated them in some detail so that they can be combined with other observations in the future.

### PATIENTS AND METHODS

Notification was obtained of all patients diagnosed between 1 January 1972, and 31 December 1974, as suffering from renal cancer in what was the Oxford Regional Hospital Board area. For each cancer patient, a control patient was selected at random, matched according to the following criteria: (1) same sex; (2) within the same 5-year age group; and (3) having had a surgical operation in the same hospital and within one month either side of that of the renal cancer patient.

If a renal cancer patient had not been operated on, the date of the most definitive diagnostic procedure was taken as equivalent to the date of operation. Patients were not accepted as controls if the control procedure was for the treatment of peptic ulceration, vascular disease or another urinary neoplasm (diseases possibly associated with coffee consumption, Roth, Ivy and Atkinson, 1944; Jick *et al.*, 1973; Cole, 1971). The conditions or procedures for which the control patients were admitted to hospital are shown in Table I. If the first control selected could not be interviewed, a second control was selected according to the same criteria as the first.

Interviews were also sought with renal cancer patients diagnosed between 1 January 1973 and 31 December 1974 who were treated by the urological service of the Royal Marsden Hospital, London, the St Peter's Group of Hospitals, London, or the Urology Department of The London Hospital. Each of these patients was matched with a control patient of the same sex and within the same 5-year age group who was registered with the same general practice as a renal cancer patient.

Each cancer and each control patient was interviewed by a research assistant who was also a trained nurse. Interviews were usually conducted in the patient's home but occasionally in hospital. Interviewing did not begin in the Oxford area until April 1973, nor in London until July 1974. The histories of the renal cancer patients were considered by the interviewer to be of doubtful reliability in 5·8% of the cases compared with 9·3% of the controls; doubts about reliability commonly applied only to the occupational history. Clinical and pathological details were taken from the patient's hospital case notes.

A total of 110 renal cancer patients in the Oxford area (47·6% of those diagnosed in the period) and 14 from the London hospitals (43·8% of those treated in the period) could not be interviewed. Of these, 86% had died before an interview could be arranged, most before we were notified of them. Nineteen of the first-selected control patients could not be interviewed (in most cases because they or their doctor refused permission) and were replaced.

TABLE I.—*Percentage Distribution of Oxford Area Control Patients According to the Condition which Led to their Admission to Hospital*

| Condition | Percentage of control patients |
|---|---|
| Other cancers | 14·9 |
| Benign tumours | 4·9 |
| Varicose veins or haemorrhoids | 5·8 |
| Abdominal hernias | 28·1 |
| Cholelithiasis | 8·3 |
| Other gastrointestinal disorders | 5·0 |
| Prostatic hypertrophy | 9·1 |
| Other genito-urinary disorders | 6·6 |
| Cystoscopy—no specific diagnosis | 5·0 |
| Other conditions | 12·4 |

### RESULTS

### *Patients' characteristics*

A total of 139 pairs of patients with and without renal cancer were interviewed. In 106 patients the site of origin of the cancer was the renal parenchyma, in 33 patients the renal pelvis. In 4 of the patients with cancer of the renal parenchyma the diagnosis was not verified histologically. Of these, one showed typical macroscopic features at post-mortem examination and the other 3 showed evidence of metastases and had typical features of the disease on intravenous pyelography (all 3 patients) or on retrograde pyelography and renal arteriography (2 patients). The remaining 102 patients with cancer of the renal parenchyma were shown to have tubular cell carcinomata (adenocarcinomata). All but one of the patients with cancer of the renal pelvis had a histological diagnosis of transitional cell carcinoma. The one exception had a squamous cell carcinoma.

Data on the age and sex of the renal cancer patients are shown in Table II. It will be noted that the patients with cancer of the renal pelvis tended to be older than those with cancer of the renal parenchyma.

The distributions of renal cancer and control patients according to their country of birth and social class are shown in Table III. There was a relative deficit of patients with cancer of the renal pelvis in social class V (1 out of 33 compared with 8 out of 33). This difference is probably a chance finding, however, as pelvic cancer shows no significant trend over the 5 socioeconomic groups ($\chi_1^2 = 2{\cdot}1, P > 0{\cdot}1$; Armitage, 1955), and national mortality data do not show any deficit of cancer of the kidney or bladder in lower social classes (Office of Population Censuses and Surveys, 1971*b*). Six of the patients with cancer of the renal parenchyma who were not born in the United Kingdom were born in either Poland or Czechoslovakia whereas the 3 matched controls born abroad were born in India, Spain and Cyprus respectively.

No appreciable differences were observed between the marital status (single, married, separated or divorced) or ethnic group distributions of the cancer patients and their controls.

TABLE II.—*Age and Sex of Renal Cancer Patients Included in the Study*

| | Oxford | | London | |
|---|---|---|---|---|
| | Parenchyma | Pelvis | Parenchyma | Pelvis |
| Men | | | | |
| No. of patients | 67 | 17 | 7 | 5 |
| Age (years): | | | | |
| Mean | 59·6 | 64·2 | 63·4 | 64·0 |
| Range | 26–83 | 51–78 | 50–76 | 48–73 |
| Women | | | | |
| No. of patients | 28 | 9 | 4 | 2 |
| Age (years): | | | | |
| Mean | 60·4 | 68·7 | 60·2 | 76·0 |
| Range | 45–84 | 59–79 | 51–72 | 70–82 |

TABLE III.—*Distributions of Renal Cancer and Control Patients According to their Country of Birth and Social Class*

| | Cancer site | | | |
|---|---|---|---|---|
| | Parenchyma | | Pelvis | |
| | Cases | Control | Cases | Control |
| Birthplace | | | | |
| United Kingdom | 98 | 103 | 32 | 31 |
| Other | 8 | 3 | 1 | 2 |
| Social class* | | | | |
| I | 8 | 5 | 0 | 2 |
| II | 19 | 19 | 10 | 5 |
| III | 55 | 53 | 15 | 15 |
| IV | 17 | 22 | 7 | 3 |
| V | 7 | 7 | 1 | 8 |

* Classified from current or pre-retirement occupation, married women classified by husband's occupation; Office of Population, Censuses and Surveys (1971*a*).

*Other diseases of the urinary system*

There were no significant differences between either group of renal cancer patients and their controls in the proportions who had suffered previously from kidney or bladder stones, haematuria or prostatic disease. There was an excess of controls over patients with cancer of either site who reported having had other urinary system disease (16 out of 139 compared with 28 out of 139; $\chi_1^2 = 4{\cdot}1$, McNemar's test, Pike and Morrow, 1970). This excess was not due to urinary system infection (reported by 9 cases and 9 controls), but mainly to a variety of disorders for which only a symptomatic diagnosis could be given (for example frequency of micturition, urinary incontinence, nocturia, " kidney pain " etc. reported by 6 cases and 14 controls). The control patients were aware that the study was into the causes of " kidney disease " and the difference could well be an artefact due to differential recall.

There were no statistically significant differences between cancer patients and controls in the proportions who had previously had either intravenous pyelography, cystoscopy or retrograde pyelography.

*Coffee and other beverages*

The distributions of renal cancer and control patients according to their consumption of coffee are shown in Table IV. There was no significant positive association between coffee consumption and renal cancer at either site. On the contrary, in the case of renal pelvic cancer there was a significant excess of patients who had never consumed coffee regularly compared with their matched controls (11 compared with 2; $\chi_1^2=7{\cdot}1, P < 0{\cdot}01$).

The estimated relative risk of renal parenchymal cancer in those currently drinking coffee daily, compared with those not drinking it daily, was 1·15 with 95% confidence limits 0·51–2·65 (Miettinen, 1970). The same estimate for renal pelvic cancer was 0·11 with 95% confidence limits 0·00–0·80. Among those who had ever drunk coffee, there were no significant differences between cases and controls in the proportions having ever used ground coffee beans (10–18%), instant coffee (77–90%), or coffee essence (5–13%).

There were no appreciable differences between cancer patients and controls in the proportions drinking various amounts of tea, or who ever drank chocolate-containing beverages, malted milk or other caffeine-free hot beverages. There were also no statistically significant differences between them in the proportions drinking various amounts of beer, wine or spirits.

TABLE IV.—*Distributions of Renal Cancer and Control Patients According to their Coffee Consumption*

| | Cups per day | | | | |
|---|---|---|---|---|---|
| Cancer site | None* | <1 | 1–2 | 3–4 | 5+ |
| Parenchyma | | | | | |
| Men | | | | | |
| Cases | 16 | 22 | 22 | 6 | 8 |
| Controls | 18 | 15 | 21 | 9 | 11 |
| Women | | | | | |
| Cases | 4 | 6 | 11 | 8 | 3 |
| Controls | 4 | 5 | 18 | 4 | 1 |
| Pelvis | | | | | |
| Men | | | | | |
| Cases | 8 | 2 | 9 | 3 | 0 |
| Controls | 3 | 7 | 8 | 3 | 1 |
| Women | | | | | |
| Cases | 4 | 1 | 5 | 1 | 0 |
| Controls | 1 | 1 | 9 | 0 | 0 |

* Includes 4 renal cancer patients and 4 controls (all men) who had given up drinking coffee.

*Tobacco use*

Renal cancer and control patients are compared with respect to tobacco use in Tables V and VI. The cigarette smoking habits of the patients with renal parenchymal cancer were not significantly different from those of their matched controls. It should be noted, however, that patients admitted to hospital with a smoking-related disease were not specifically excluded from the control group, and there were 2 men with bronchial cancer among the controls, both of whom smoked more than 60 cigarettes daily. The estimated relative risk of cancer of the renal parenchyma in current cigarette smokers, compared with non-smokers is 1.06 in men (37 discordant pairs, case a current smoker in 19, control in 18) and 1·00 in women, (14 discordant pairs, case a current smoker in 7, control in 7), with 95% confidence limits 0·52–2·16 and

TABLE V.—*Distributions of Renal Cancer and Control Patients According to their Cigarette-smoking Habits*

| | | | Current smokers cigarettes/day | | |
|---|---|---|---|---|---|
| Cancer site | "Never" smokers | Ex-smokers | <20 | 20–29 | 30+ |
| Parenchyma | | | | | |
| Men | | | | | |
| Cases | 11 | 30 | 13 | 13 | 7 |
| Controls | 12 | 30 | 17 | 9 | 6 |
| Women | | | | | |
| Cases | 15 | 7 | 7 | 2 | 1 |
| Controls | 13 | 9 | 8 | 2 | 0 |
| Pelvis | | | | | |
| Men | | | | | |
| Cases | 1 | 8 | 4 | 5 | 4 |
| Controls | 5 | 8 | 5 | 3 | 1 |
| Women | | | | | |
| Cases | 8 | 3 | 0 | 0 | 0 |
| Controls | 5 | 3 | 3 | 0 | 0 |

TABLE VI.—*Distributions of Male Renal Cancer and Control Patients According to their Use of Pipes, Cigars and Chewing Tobacco or Snuff*

| Cancer site | Frequency of use: Never | Ex- | Occasional | Regular |
|---|---|---|---|---|
| Parenchyma | | | | |
| | | | Pipes | |
| Cases | 35 | 18 | 12 | 9 |
| Controls | 29 | 19 | 11 | 15 |
| | | | Cigars | |
| Cases | 41 | 2 | 27 | 4 |
| Controls | 27 | 6 | 37 | 4 |
| | | | Chewing tobacco or snuff | |
| Cases | 64 | 5 | 4 | 1 |
| Controls | 63 | 8 | 2 | 1 |
| Pelvis | | | | |
| | | | Pipes | |
| Cases | 11 | 5 | 6 | 0 |
| Controls | 11 | 5 | 4 | 2 |
| | | | Cigars | |
| Cases | 13 | 2 | 7 | 0 |
| Controls | 9 | 2 | 11 | 0 |
| | | | Chewing tobacco or snuff | |
| Cases | 20 | 1 | 1 | 0 |
| Controls | 19 | 0 | 1 | 2 |

0·30–3·37 respectively. The estimate in men does not change if the controls with bronchial cancer are excluded because the renal cancer patients matched with them were also current smokers.

There is a marked trend towards heavier cigarette consumption by men with cancer of the renal pelvis compared with their controls, which is statistically significant at the 5% level ($\chi_1{}^2$ for trend = 4·99, over the 4 categories "never" smokers and smokers of $< 20$, 20–29 and 30 + cigarettes per day). The estimated relative risk of cancer of the renal pelvis in male current smokers, compared with non-smokers, is 1·80 with 95% confidence limits 0·54–6·84 (14 discordant pairs, case a current smoker in 9, control in 5). Of the men with pelvic cancer who had ever smoked, 76·2% inhaled the cigarette smoke, compared with 64·7% in the controls.

There was no excess of male cancer patients compared with controls who used tobacco in forms other than cigarettes (Table VI). The proportion of men with renal parenchymal cancer who had ever used cigars was, in fact, substantially less than in their controls (33 of 74 compared with 47 of 74; $\chi_1{}^2 = 5{\cdot}3$).

*Saccharin consumption*

The proportions of patients who had ever used saccharin tablets for at least 6 months at various levels of consumption are shown in Table VII. No appreciable differences were observed, nor were any observed in the proportions consuming cordials or aerated waters which commonly contain saccharin, nor in the proportions consuming different amounts of these beverages. No patient made reference to the use of artificial sweeteners other than saccharin.

TABLE VII.—*Distributions of Renal Cancer and Control Patients According to their Maximum Ever Consumption of Saccharin Tablets**

| Cancer site | Tablets per day: Never | <4 | 5–9 | 10+ |
|---|---|---|---|---|
| Parenchyma | | | | |
| Cases | 86 | 10 | 8 | 2 |
| Controls | 88 | 8 | 9 | 1 |
| Pelvis | | | | |
| Cases | 27 | 2 | 3 | 1 |
| Controls | 28 | 1 | 2 | 2 |

* For more than 6 months.

*Medications*

The distributions of renal cancer and control patients according to the maximum level at which they had ever taken analgesic tablets for more than six months are shown in Table XIII. There was a highly significant excess of patients with parenchymal cancer—but not of patients with pelvic cancer—who had taken analgesics daily (15 of 106 compared with 2 of 106; $P < 0{\cdot}005$). All but 2 of the cancer patients who had taken analgesics daily were taking them at the time of diagnosis.

TABLE VIII.—*Distributions of Renal Cancer and Control Patients According to their Maximum Ever Consumption of Analgesic Tablets**

| Cancer site | Never | < Once/month | < Once/week | < Once/day | Daily |
|---|---|---|---|---|---|
| | Maximum frequency of consumption of analgesic tablets* | | | | |
| Parenchyma | | | | | |
| Cases | 40 | 24 | 15 | 12 | 15† |
| Controls | 56 | 28 | 9 | 11 | 2 |
| Pelvis | | | | | |
| Cases | 15 | 12 | 2 | 4 | 0 |
| Controls | 18 | 4 | 4 | 4 | 3 |

* For a period of 6 months or more. Each succeeding frequency category is exclusive of the preceding categories.
† $\chi_1^2 = 9 \cdot 6$, $P < 0 \cdot 005$, for difference between cases and controls.

The 15 patients with cancer of the renal parenchyma who took analgesic tablets daily had taken between 1 and 12 tablets daily for periods of up to 30 years. Six had taken analgesics for only one year or less before the diagnosis of their cancer, while 7 had taken them for 5 years or more. There was no preponderant analgesic type. Most patients were currently taking a single-component analgesic such as codeine, paracetamol or aspirin; only 4 were taking preparations which had formerly contained phenacetin.

The use of sulphonamide antibacterial drugs was also recorded. Thirty-one (22·3%) of the renal cancer patients had ever been treated with them, compared with 27 (19·4%) of the controls. The proportion was essentially the same in patients with renal pelvic cancer (21·2%) as in patients with renal parenchymal cancer (22·1%). None of the patients had received long-term sulphonamide therapy for the prophylaxis of rheumatic fever and less cancer patients than controls had ever received long-term treatment for urinary infection (5 compared with 12). Only one patient had ever taken the contraceptive pill.

*Animal protein consumption*

The distributions of the renal cancer and control patients according to their frequency of consumption of meat, poultry, seafood, eggs, milk and cheese are shown in Tables IX and X. There were no appreciable differences between the patients with parenchymal cancer and their controls in any of these. Patients with pelvic cancer, however, showed a significant trend towards a lower consumption of cheese ($\chi_1^2$ for trend $= 4 \cdot 93$, $P < 0 \cdot 05$) and a somewhat lower consumption of eggs.

*Occupation*

Patients were questioned specifically regarding occupational exposure to substances known to be associated with bladder cancer or suspected of being associated with renal cancer. The substances to which an appreciable number of patients had been exposed for at least one year and the proportions exposed are shown in Table XI. No significant differences were found. In particular, we were unable to confirm Schmauz and Cole's (1974) finding of an excess of cancer of the renal pelvis in leather workers, although the study area included Northamptonshire where boot and shoe manufacture is common and where there is an excess of nasal cancer in leather workers (Acheson, Cowdell and Jolles, 1970).

### DISCUSSION

The interpretation of our results is complicated by the large number of comparisons that have been made and

TABLE IX.—*Distributions of Patients with Cancer of the Renal Parenchyma and Control Patients According to their Consumption of Animal Protein*

| | Frequency of consumption* | | | | |
|---|---|---|---|---|---|
| | | | Meals/day | | |
| Meat | < Once/week | < Once/day | 1 | 2 | 3 |
| Cases | 0 | 40 | 43 | 21 | 2 |
| Controls | 1 | 39 | 48 | 16 | 2 |
| Poultry | < Once/month | < Once/week | < Once/day | Daily | |
| Cases | 27 | 45 | 34 | 0 | |
| Controls | 21 | 51 | 34 | 0 | |
| Seafood | < Once/month | < Once/week | < Once/day | Daily | |
| Cases | 13 | 20 | 73 | 0 | |
| Controls | 17 | 23 | 66 | 0 | |
| | | | | No./day | |
| Eggs | < One/month | < One/week | < One/day | 1 | 2 + |
| Cases | 5 | 6 | 57 | 32 | 6 |
| Controls | 7 | 6 | 62 | 21 | 10 |
| | | | | Glasses/day | |
| Milk | Never | < One glass/week | < One glass/day | 1 | 2 + |
| Cases | 45 | 10 | 16 | 14 | 21 |
| Controls | 47 | 11 | 7 | 22 | 19 |
| | | | | Ounces/day | |
| Cheese | < 4 oz/month | < 4 oz/week | < 1 oz/day | 1–2 | 3 + |
| Cases | 8 | 17 | 29 | 45 | 7 |
| Controls | 9 | 20 | 33 | 36 | 8 |

* Each succeeding frequency category is exclusive of the preceding categories.

TABLE X.—*Distributions of Patients with Renal Pelvic Cancer and Control Patients According to their Consumption of Animal Protein*

| | Frequency of consumption* | | | | |
|---|---|---|---|---|---|
| | | | Meals/day | | |
| Mean | < Once/week | < Once/day | 1 | 2 | 3 |
| Cases | 2 | 14 | 14 | 3 | 0 |
| Controls | 0 | 16 | 16 | 1 | 0 |
| Poultry | < Once/month | < Once/week | < Once/day | Daily | |
| Cases | 9 | 12 | 12 | 0 | |
| Controls | 5 | 19 | 9 | 0 | |
| Seafood | < Once/month | < Once/week | < Once/day | Daily | |
| Cases | 1 | 7 | 25 | 0 | |
| Controls | 5 | 5 | 23 | 0 | |
| | | | | No./day | |
| Eggs | < One/month | < One/week | < One/day | 1 | 2 + |
| Cases | 2 | 1 | 25 | 4 | 1 |
| Controls | 1 | 2 | 18 | 7 | 5 |
| | | | | Glasses/day | |
| Milk | Never | < One glass/week | < One glass/day | 1 | 2 + |
| Cases | 17 | 2 | 3 | 9 | 2 |
| Controls | 16 | 2 | 3 | 9 | 3 |
| | | | | Ounces/day | |
| Cheese | < 4 oz/month | < 4 oz/week | < 1 oz/day | 1–2 | 3 + |
| Cases | 4 | 11 | 10 | 8 | 0 |
| Controls | 1 | 6 | 12 | 14 | 0 |

* Each succeeding frequency category is exclusive of the preceding categories.

TABLE XI.—*Numbers of Renal Cancer and Control Patients Having Ever Had Occupational Exposure to Various Compounds**

| Cancer site | Dyes | Leather | Rubber | Lead | Paint |
|---|---|---|---|---|---|
| Parenchyma | | | | | |
| Cases | 6 | 7 | 5 | 12 | 13 |
| Controls | 14 | 15 | 3 | 11 | 13 |
| Pelvis | | | | | |
| Cases | 3 | 1 | 0 | 3 | 4 |
| Controls | 1 | 1 | 3 | 3 | 3 |

* For periods of 1 year or more.

the paucity of the data. Not only may some differences of apparently low probability have occurred by chance but any measure of association that is obtained will have fairly wide confidence limits. For example, the relative risk of renal parenchymal cancer in those drinking coffee regularly was estimated at 1·15 with 95% confidence limits 0·51–2·65. These data could therefore be reasonably considered to be consistent with a relative risk of about 2. In these situations a subjective decision must be made, on the basis of all available information, either to reject the prior hypothesis (either the "null" hypothesis or an hypothesis suggesting a particular association) or to attribute the results to chance.

Subject to the above reservations, this study suggests that there is no positive association between coffee consumption and renal cancer in individuals. Wynder *et al.* (1974) also found no evidence of such an association. Schmauz and Cole (1974) found a positive relationship between coffee consumption and cancers of the renal pelvis and ureter in a small series of men with one or the other of these diseases. Our study does not support this observation (in fact there was a negative association between cancer of the renal pelvis and coffee consumption); but none of our patients with renal pelvic cancer, nor their controls, drank 7 or more cups of coffee per day, the level of consumption which appeared to confer a disproportionate degree of risk in Schmauz and Cole's study.

The data presented in Table V confirm the finding of Schmauz and Cole (1974) of a positive association between cigarette smoking and cancer of the renal pelvis in men. This is not surprising in view of the close clinical association between cancer of the renal pelvis and cancer of the bladder, and other evidence that they may share aetiological factors (Poole-Wilson, 1969). In contrast, the data on cancer of the renal parenchyma suggest that this disease is not associated positively with tobacco use (Tables V and VI). The upper 95% confidence limit of the relative risk estimate for cigarette smoking in men (2·16) is incompatible with the degree of positive association found by Bennington and Laubscher (1968), (relative risk about 5). It is compatible, however, with the degree of positive association found by Wynder *et al.* (1974) (relative risk in men 2·0), who also found evidence of a dose-effect relationship. Similar results have been obtained for renal cancer as a whole in the prospective studies of mortality in cigarette smokers (relative risk varying from 1·5 to 3·9; Hammond, 1966; Kahn, 1966; Doll and Peto, unpublished data).

An unexpected finding of this study which may not be due to chance ($P < 0{\cdot}005$) is the positive association between cancer of the renal parenchyma and the daily consumption of analgesics. There was no evidence of the reported association between analgesic consumption and cancer of the renal pelvis (Johansson *et al.*, 1974) but this may not be surprising as papillary necrosis associated with analgesic abuse, which is usually present in analgesic-associated cases of renal pelvic cancer, is relatively rare in the United Kingdom (Davies, Kennedy and Roberts, 1970).

It is difficult to reach a conclusion regarding the possible causality of the positive association between analgesic consumption and cancer of the renal parenchyma. The number of tablets taken was generally higher among the renal cancer patients than the controls, but they had been taken for one year or less by 6 of the 15 exposed cancer patients. The preparations used were quite diverse and only 4 renal cancer patients (and one control) used preparations which formerly contained phenacetin. Alternatively, the prodromal symptoms of renal cancer may have led to the use of analgesics. This could account for the use of analgesics for only a short period before diagnosis. Such a non-causal explanation seems the most likely because no excess of renal parenchymal cancer has been reported from the follow-up of patients with analgesic nephropathy (Bengtsson *et al.*, 1968; Høybye and Nielsen, 1971; Taylor, 1972).

There was no evidence in this study of a positive association between cancer of the renal parenchyma and the consumption of animal protein. Although the method of categorizing consumption of the various forms of animal protein was rather crude, this form of categorization, at least for meat and eggs, has been shown to correlate well with serum vitamin $B_{12}$ levels (Armstrong *et al.*, 1974). The results of this study are therefore probably incompatible with the degree of positive association between animal protein consumption and renal cancer suggested by their geographical correlation (Armstrong and Doll, 1975). It may be that the positive geographical association is secondary to a positive association between these 2 variables and some other factors, for example fat or cholesterol consumption (Wynder *et al.*, 1974).

Cancer of the renal pelvis showed a weakly negative association with several forms of animal protein consumption which was statistically significant for cheese consumption ($P < 0{\cdot}05$). Although this may have been due to chance, it raises the possibility that either cheese or perhaps animal protein in general may protect against the development of renal pelvic cancer by, for example, increasing the rate of metabolism of carcinogens (McLean and Magee, 1970; Paine and McLean, 1973; McLean, 1973).

We gratefully acknowledge the assistance of Miss C. Hunt and the staff of the Oxford Regional Cancer Registry, and of the many consultants and general practitioners who willingly permitted us to interview their patients.

### REFERENCES

ACHESON, E. D., COWDELL, R. H. & JOLLES, B. (1970) Nasal Cancer in the Northamptonshire Boot and Shoe Industry. *Br. med. J.*, i, 385.

ARMITAGE, P. (1955) Tests for Linear Trends in Proportions and Frequencies. *Biometrics*, **11**, 375.

ARMSTRONG, B. K., DAVIS, R. E., NICOL, D. J., VAN MERWYK, A. J. & LARWOOD, C. J. (1974) Hematological, Vitamin B12, and Folate Studies on Seventh-day Adventist Vegetarians. *Am. J. clin. Nutr.*, **27**, 712.

ARMSTRONG, B. & DOLL, R. (1975) Environmental Factors and Cancer Incidence and Mortality in Different Countries with Special Reference to Dietary Practices. *Int. J. Cancer.*, **15**, 617.

BENGTSSON, U., ANGERVALL, L., EKMAN, H. & LEHMANN, L. (1968) Transitional Cell Tumours of the Renal Pelvis in Analgesic Abusers. *Scand. J. Urol. Nephrol.*, **2**, 145.

BENNINGTON, J. L. & LAUBSCHER, F. A. (1968) Epidemiologic Studies on Carcinoma of the Kidney. 1. Association of Renal Adenocarcinoma with Smoking. *Cancer, N.Y.*, **21**, 1069.

COLE, P. (1971) Coffee-drinking and Cancer of the Lower Urinary Tract. *Lancet*, i, 1335.

DAVIES, D. J., KENNEDY, A. & ROBERTS, C. (1970) The Aetiology of Renal Medullary Necrosis: A Survey of Adult Cases in Liverpool. *J. Path.*, **100**, 257.

HAMMOND, E. C. (1966) Smoking in Relation to the Death Rates of One Million Men and Women. *Natn. Cancer Inst. Monog.*, **19**, 127.

HANSEN, P. B. & BICHEL, J. (1952) Carcinogenic Effect of Sulphonamides. *Acta Radiol.*, **37**, 258.

HOYBYE, G. & NIELSEN, O. E. (1971) Renal Pelvic Carcinoma in Phenacetin Abusers. *Scand. J. Urol. Nephrol.*, **5**, 190.

JICK, H., MIETTINEN, O. S., NEFF, R. K., SHAPIRO, S., HEINONEN, O. P. & SLONE, D. (1973) Coffee and Myocardial Infarction. *New Engl. J. Med.*, **289**, 63.

JOHANSSON, S., ANGERVALL, L., BENATSSON, U. & WAHLQUIST, L. (1974) Uroepithelial Tumours of the Renal Pelvis Associated with Abuse of Phenacetin Containing Analgesics. *Cancer, N.Y.*, **33**, 743.

KAHN, H. A. (1966) The Dorn Study of Smoking and Mortality among U.S. Veterans: Report on Eight and One Half Years of Observation. *Natn. Cancer Inst. Monog.*, **19**, 1.

McLean, A. E. M. (1973) Diet and the Chemical Environment as Modifiers of Carcinogenesis. In *Host Environment Interactions and the Etiology of Cancer in Man.* Ed. R. Doll and J. Vodopija. Lyons: IARC. p. 223.

McLean, A. E. M. & Magee, P. N. (1970) Increased Renal Carcinogenesis by Dimethylnitrosamine in Protein Deficient Rats. *Br. J. exp. Path.*, **51**, 587.

Miettinen, O. S. (1970) Estimation of Relative Risk from Individually Matched Series. *Biometrics*, **26**, 75.

Office of Population Censuses and Surveys (1971*a*) *Classification of Occupations* 1971. London: HMSO.

Office of Population Censuses and Surveys (1971*b*) The Registrar General's Decennial Supplement. England and Wales, 1961. *Occupational Mortality Tables.* London: HMSO.

Paine, A. J. & McLean, A. E. M. (1973) The Effect of Dietary Protein and Fat on the Activity of Amyl Hydrocarbon Hydrolase in Rat Liver, Kidney and Lung. *Biochem. Pharmac.*, **22**, 2875.

Pike, M. C. & Morrow, R. H. (1970) Statistical Analysis of Patient-Control Studies in Epidemiology. Factor under Investigation an All-or-None Variable. *Br. J. prev. soc. Med.*, **24**, 42.

Poole-Wilson, D. S. (1969) Occupational Tumours of the Renal Pelvis and Ureter arising in the Dye-making Industry. *Proc. R. Soc. Med.*, **62**, 93.

Roth, J. A., Ivy, A. C. & Atkinson, A. J. (1944) Caffeine and Peptic Ulcer. *J. Am. med. Ass.*, **126**, 814.

Schmauz, R. & Cole, P. (1974) Epidemiology of Cancer of the Renal Pelvis and Ureter. *J. natn. Cancer Inst.*, **52**, 1431.

Shennan, D. H. (1973) Renal Carcinoma and Coffee Consumption in 16 Countries. *Br. J. Cancer*, **28**, 473.

Taylor, J. S. (1972) Carcinoma of the Urinary Tract and Analgesic Abuse. *Med. J. Aust.*, **1**, 407.

Van Esch, G. J., Van Genderen, H. & Vink, H. H. (1962) The Induction of Renal Tumours by Feeding of Basic Lead Acetate to Rats. *Br. J. Cancer*, **16**, 289.

Wynder, E. L., Mabuchi, K. & Whitmore, W. F. (1974) Epidemiology of Adenocarcinoma of the Kidney. *J. natn. Cancer Inst.*, **53**, 1619.